# AI-Guided Delineation of Gross Tumor Volume for Body Tumors: A Systematic Review

**DOI:** 10.3390/diagnostics15070846

**Published:** 2025-03-26

**Authors:** Lea Marie Pehrson, Jens Petersen, Nathalie Sarup Panduro, Carsten Ammitzbøl Lauridsen, Jonathan Frederik Carlsen, Sune Darkner, Michael Bachmann Nielsen, Silvia Ingala

**Affiliations:** 1 Department of Diagnostic Radiology, Copenhagen University Hospital Rigshospitalet, 2100 Copenhagen, Denmark; 2 Department of Clinical Medicine, University of Copenhagen, 2100 Copenhagen, Denmark; 3 Department of Computer Science, University of Copenhagen, 2100 Copenhagen, Denmark; 4 Department of Oncology, Rigshospitalet, 2100 Copenhagen, Denmark; 5 Radiography Education, University College Copenhagen, 2200 Copenhagen, Denmark; 6 Cerebriu A/S, 1434 Copenhagen, Denmark; 7 Department of Diagnostic Radiology, Copenhagen University Hospital Herlev and Gentofte, 2730 Herlev, Denmark

**Keywords:** gross tumor volume, segmentation, artificial intelligence

## Abstract

**Background**: Approximately 50% of all oncological patients undergo radiation therapy, where personalized planning of treatment relies on gross tumor volume (GTV) delineation. Manual delineation of GTV is time-consuming, operator-dependent, and prone to variability. An increasing number of studies apply artificial intelligence (AI) techniques to automate such delineation processes. **Methods**: To perform a systematic review comparing the performance of AI models in tumor delineations within the body (thoracic cavity, esophagus, abdomen, and pelvis, or soft tissue and bone). A retrospective search of five electronic databases was performed between January 2017 and February 2025. Original research studies developing and/or validating algorithms delineating GTV in CT, MRI, and/or PET were included. The Checklist for Artificial Intelligence in Medical Imaging (CLAIM) and Transparent Reporting of a multivariable prediction model for Individual Prognosis Or Diagnosis statement and checklist (TRIPOD) were used to assess the risk, bias, and reporting adherence. **Results**: After screening 2430 articles, 48 were included. The pooled diagnostic performance from the use of AI algorithms across different tumors and topological areas ranged 0.62–0.92 in dice similarity coefficient (DSC) and 1.33–47.10 mm in Hausdorff distance (HD). The algorithms with the highest DSC deployed an encoder–decoder architecture. **Conclusions**: AI algorithms demonstrate a high level of concordance with clinicians in GTV delineation. Translation to clinical settings requires the building of trust, improvement in performance and robustness of results, and testing in prospective studies and randomized controlled trials.

## 1. Introduction

Approximately 50% of all oncologic patients undergo radiation therapy, making this one of the cornerstones of cancer treatment. Personalized planning of radiation therapy relies on anatomical tumor delineation in a scan prior to treatment, referred to as Gross Tumor Volume (GTV) [1]. However, manual GTV delineation is a time-consuming and operator-dependent procedure prone to intra- and interobserver variability [2]. While the development and refinement of artificial intelligence (AI)-based applications for automated segmentation of tumors promise to automate this task, making it both faster and more objective, applications in real-life clinical scenarios are still lacking due to insufficient evidence on their generalizability [3].

The performance of algorithms is linked to several factors, including the modality employed for GTV delineation, labeling, annotation, and segmentation protocols, as well as quality assurance. It is widely acknowledged that data quality plays a pivotal role in shaping the performance of AI algorithms and the generalizability of their results. Specifically, the fitness of both the quantity and quality of the data for the specific problem at hand significantly influences algorithm performance. Additionally, the architecture and backbone of the proposed model can also widely affect the outcome [3].

To date, no comprehensive review of AI-based algorithms to delineate GTV for radiotherapy purposes in body tumors (thorax, abdomen, and associated soft tissue and bone) has been published. Former segmentation challenges for head and neck cancers have provided extensive overviews of the applications and available literature in their respective domains. With this in mind, we aimed to conduct a systematic review of the available literature on existing algorithms by topological tumor location and assess their performance based on accuracy, robustness, efficiency, generalizability, and interpretability. This was conducted in order to evaluate AI-based method performance for delineation of the GTV compared to manual delineation for tumors eligible for radiation therapy in body tumors.

## 2. Materials and Methods

Institutional review board approval of this study was deemed unnecessary as the data utilized in this study were retrospectively sourced exclusively from publicly available databases, and no direct involvement or handling of human subjects occurred.

Search strategy:

To identify potentially relevant articles, a title/abstract/keyword search was performed in PubMed, Scopus, Cochrane Library, IEEE, and Web of Science. The search string used was “(GTV OR gross tumor volume) AND (segmentation OR delineation)”. The search was restricted to peer-reviewed original research articles in English published between 2017 and 2025, both years included. The literature search was completed on 4th February 2025.

Inclusion and exclusion criteria: The Preferred Reporting Items for Systematic Reviews and Meta-Analyses (PRISMA) guidelines for literature search and study selection were followed [4]. After the removal of duplicates, the titles and abstracts of the articles were screened independently by two authors (L.M.P, N.S.P.). Inclusion criteria were (1) original research peer reviewed articles; (2) focused on the automatic segmentation of GTV of any type of cancer eligible for radiation therapy in the thorax, esophagus, abdomen, pelvis, soft tissue, and bone; and (3) utilizing one or more imaging modalities including computed tomography (CT), magnetic resonance imaging (MRI), and/or positron emission tomography (PET) for GTV segmentations. Reasons for exclusion included phantom data, animal studies, studies only using publicly available datasets to ensure diverse patient cohorts, and GTV obtained from any other modalities than CT, PET, or MRI, as well as studies focused on head and neck, brain, pediatric patients, or conference papers/abstracts.

Evaluation markers and quality assessment: For each publication, the name of the first author, year of publication, modality, dice similarity coefficient (DSC), Hausdorff distance (HD), cancer type, sample size, model, backbone, and numbers of specialized personal delineating the GTV were extracted for further analysis. The risk, bias, and reporting adherence were assessed using the Checklist for Artificial Intelligence in Medical Imaging (CLAIM) as well as the Transparent Reporting of a multivariable prediction model for Individual Prognosis Or Diagnosis statement and checklist (TRIPOD) [5,6].

To prevent unfair comparisons in study outcomes due to different selection of (secondary) outcome measures, only the best performance as measured by the DSC and HD for the validation cohort was assessed. The varying outcomes of the publications may be attributed to modifications in the hyperparameters or test of different algorithms.

## 3. Results

**Figure 1:**
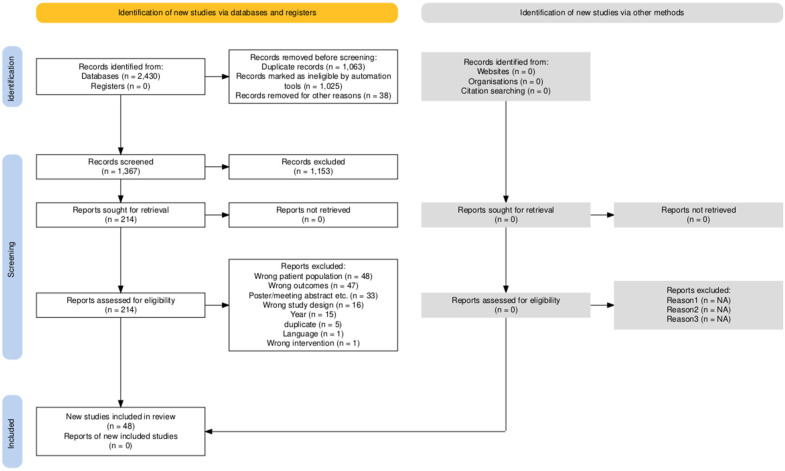
Preferred Reporting Items for Systematic Reviews and Meta-Analysis (PRISMA).

In brief, 2430 records were initially identified. From these, 1063 were duplicates and hence removed. Afterwards, 1367 abstracts were screened and 1153 did not fulfill the inclusion criteria. A full-text review was performed for 214 records and 166 of these were then excluded based on the topological location of the tumor (e.g., brain tumors), study outcomes and/or design beyond the scope of this review, year of publication preceding 2017, and presence of duplicates. As a result, a total of 48 publications were included in this review. A flow diagram showing a schematic overview of the steps followed in this study in accordance with the Preferred Reporting Items for Systematic reviews and Meta-Analyses (PRISMA) guidelines is shown in Figure 1 [4]. The publications reported in the results section were reviewed based on the affected topological district, listed in Table 1, Table 2, Table 3 and Table 4, and split based on the location and tumor type.

### 3.1. Thorax

An overview of the studies addressing GTV delineation in the thoracic cavity included in this review (n = 15) is provided in Table 1 [7,8,9,10,11,12,13,14,15,16,17,18,19,20,21]. All studies focused on lung tumors, however, only eight specified the histological subtype, which was non-small cell lung cancer (NSCLC) for all of them [7,8,10,12,14,17,20,21]. Yu X. et al. narrowed down the study scope to a specific stage (NSCLC stage III) and Kunkyab T. et al. described the baseline characteristics including tumor type and location [7,17]. There were no studies available regarding the delineation of small cell lung cancer, mesothelioma, and mediastinal tumors (thymoma, thymic carcinoma, lymphoma, germ cell and neurogenic tumors, or primary cardiac tumors).

Kunkyab T. et al. achieved the highest overall DSC and one of the lowest HD values (DSC = 0.92, HD = 1.33). Their study utilized CT scans from two publicly available datasets as well as their internal clinical database (BC Cancer Kelowna), comprising 676 cases (train: 563, test: 113) [7]. The dataset covered adenocarcinoma, squamous cell carcinoma, NSCLC (not specified), and large cell carcinoma. The ML architecture employed was based on a dual-branch encoder, with a deep 3D convolutional neural network (CNN) extracting semantic features from low-resolution data, while a shallow 3D CNN preserves positional details from high-resolution inputs. They then utilize a multi-scale feature pyramid network to enhance feature extraction. A transformer module integrates deformable self-attention for efficient region focus, feed-forward networks for feature transformation, and skip connections to mitigate gradient vanishing. The decoder reconstructs the segmentation map using CNN-based up-sampling, residual blocks, and encoder–decoder skip connections to retain fine-grained details. Additionally, they provide inference time (Co-ReTr = 12.03 s) and segmentation performance across multiple tumors (DSC = 0.89, HD = 1.66).

In line with clinical standards for assessment of tumor size [22], CT was the preferred imaging modality for GTV delineation in the thoracic cavity across all the publications analyzed, over 73% of studies relying solely on CT [7,9,10,11,12,13,16,17,18,19,21], and approximately 27% employing it in conjunction with PET [8,14,15,20]. The mean sample size across all publications with regards to the thorax was N = 216, (range: 9–871).

All 15 publications openly disclosed their data sources, suggesting a commitment to transparency. The data split method was reported in over half of the publications (60%). Five papers reported using an external testing set [7,10,11,19,21]. Notably, 93% of the publications provided information regarding the staff involved and their educational qualifications in the delineation of the ground truth [7,8,9,10,11,12,13,14,15,16,17,18,19,21].

**Figure 2:**
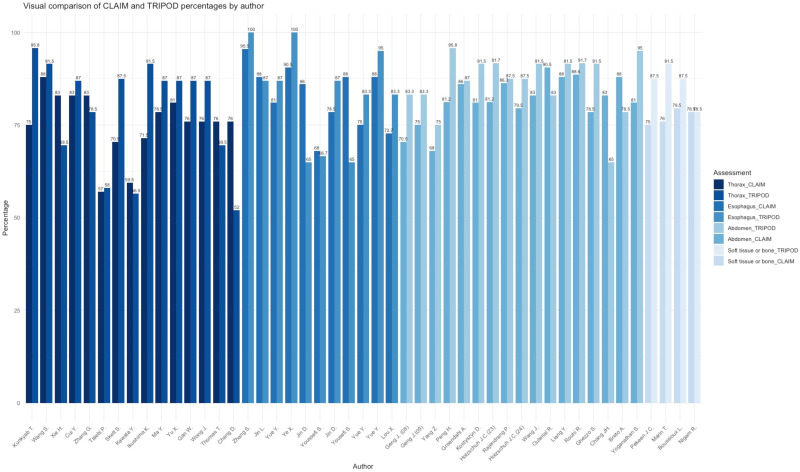
Assessment of the Checklist for Artificial Intelligence in Medical Imaging (CLAIM) and Transparent Reporting of a multivariable prediction model for Individual Prognosis Or Diagnosis (TRIPOD) checklist adherence [7,8,9,10,11,12,13,14,15,16,17,18,19,20,21,23,24,25,26,27,28,29,30,31,32,33,34,35,36,37,38,39,40,41,42,43,44,45,46,47,48,49,50,51,52,53,54].

**Figure 3:**
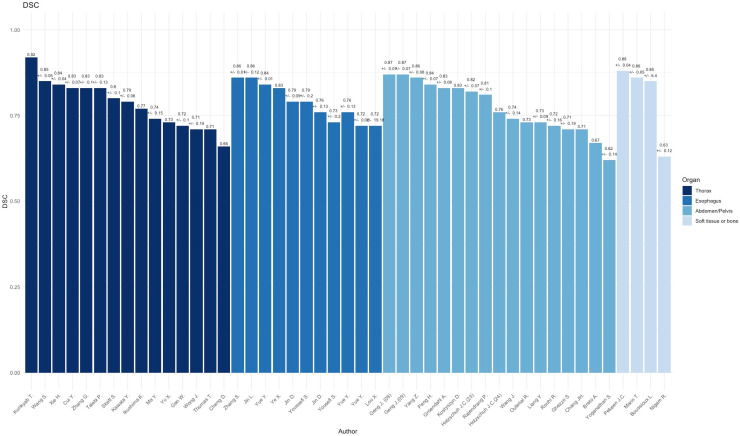
Reported dice similarity coefficient (DSC) overview for all included publications [7,8,9,10,11,12,13,14,15,16,17,18,19,20,21,23,24,25,26,27,28,29,30,31,32,33,34,35,36,37,38,39,40,41,42,43,44,45,46,47,48,49,50,51,52,53,54].

**Figure 4:**
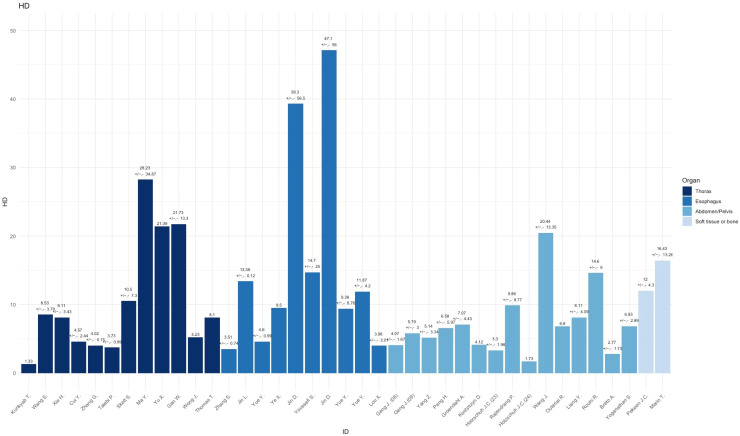
Reported Hausdorff distance (HD) overview from all publications [7,8,9,10,11,12,13,16,17,18,19,20,23,24,25,26,27,28,29,31,32,33,34,35,36,37,38,39,40,41,42,43,44,45,46,49,50,51,52].

Compared to other topographical areas, the thorax subset had the lowest adherence to the CLAIM and TRIPOD guidelines, with an average of 75.6% for CLAIM and 70.0% for TRIPOD, resulting in an overall adherence of 77.3%. Figure 2 summarizes the assessment of TRIPOD and CLAIM adherence. These results indicate that studies focusing on the thorax tend to have lower compliance with the reporting standards, signaling room for improvement for both TRIPOD and CLAIM adherence. The mean DSC outcomes of the examined publications was 0.78 with an interquartile range (IQR) of 0.10 (range: 0.66–0.92) and the HD measurements derived had a mean of 10.5 mm, and an IQR of 8.79 mm (range: 1.33–28.23 mm). Figure 3 and Figure 4 summarize the DSC and HD outcomes.

### 3.2. Esophagus

An overview of the studies addressing GTV delineation in the esophagus (n = 11) is provided in Table 2 [23,24,25,26,27,28,29,30,31,32,33]. All publications focused on esophageal cancer, however, five publications specified in greater characteristics of the dataset [23,24,26,27,33]. Six of the papers utilized both PET and CT [25,26,27,29,31,32], whereas the remaining five papers solely relied on CT [23,24,28,30,33].

Zhang S. et al. achieved the highest DSC (0.869 ± 0.006) and lowest HD (3.51 ± 0.74) in a multi-cohort study with 580 patients [23]. The study provided detailed patient characteristics, including staging, tumor location, volume in cm^3^, and length in mm. The ground truth generated based on consensus from two board-certified radiation oncologists and eight board-certified radiologists across four hospitals was tested against an AI model using the 3D nnU-Net architecture.

Approximately 72% of the publications explicitly documented the origin of their data. Three publications declared using both internal and external validation datasets and notably also had the largest datasets [23,26,28]. Cross-validation was implemented in roughly 60% of the papers [25,26,27,29,32]. Nine out of the eleven papers disclosed the personnel responsible for delineating the ground truth data [23,24,25,26,27,29,31,32,33].

The esophagus subset showed higher adherence, averaging 82.8% for CLAIM and 83.6% for TRIPOD, leading to an overall adherence of 84.2% (Figure 2) This suggests a high level of compliance with both reporting guidelines, indicating that studies related to the esophagus generally meet transparency and accuracy standards. The mean sample size of included samples across all papers was N = 238, (range: 49–606). The mean DSC outcome of the examined publications was 0.78 with an IQR of 0.09 (range: 0.72–0.86) and the HD measurements derived had a mean of 15.70 mm, and an IQR of 8.58 mm (range: 4.60–47.10 mm). Figure 3 and Figure 4 summarize the DSC and HD outcomes.

**Table 2:** Esophagus publications.

Author (Year)	Modality	DSC/HD	Cancer Type (N)	Model	Backbone	Delineation Staff
Zhang S. et al. [23] (2024)	CT	0.869 ± 0.006/ 3.51 ± 0.74	Esophageal cancer (580)	3D nn-U-Net	skip connections between the encoder and decoder improved the segmentation	Four oncologists and eight radiologists
Jin L. et al. [24] (2022)	CT	0.86 ± 0.12/ 13.38 ± 0.12	Esophageal cancer (215)	3D VUMix-Net	3D V-Net for localization, 2D U-Net for segmentation	One radiation oncologist
Yue Y. et al. [25] (2022)	CT, PET	0.84 + 0.009/ 4.60 ±0.99	Esophageal cancer (164)	GloD-LoATU-Net	ConV-Transformer with GloDAT and LoAT blocks	Two nuclear clinicians, one chief oncologist.
Ye X. et al. [26] (2022)	CT, PET	0.83/ 9.50	Esophageal cancer (606)	Two-Stream 3D PSNN	3D Progressive Semantically Nested Network	Two expert healthcare professionals
Jin D. et al. [27] (2021)	CT, PET	0.79 ± 0.09/ 39.30 ± 56.5	Esophageal cancer (148)	Two-Stream 3D PSNN	3D Progressive Semantically Nested Network	Two experienced radiation oncologists
Youssefi S. et al. [28] (2021)	CT	0.79 ± 0.20/ 14.7 ± 25.0	Esophageal Cancer (288)	DDAU-Net	Dilated Dense Attention U-Net	N/A
Jin D. et al. [29] (2019)	CT, PET	0.76 ± 0.13/ 47.10 ± 56.0	Esophageal cancer (110)	Two-Stream 3D PSNN	3D Progressive Semantically Nested Network	Two experienced radiation oncologists
Yousefi S. et al. [30] (2018)	CT	0.73 ± 0.20/ N/A	Esophageal cancer (49)	3D Dense U-NET	3D U-NET network with dense blocks	N/A
Yue Y. et al. [31] (2024)	CT, PET	0.76 ± 0.13/ 9.38 ± 8.76	Esophageal Cancer (164)	TransAttPSNN	Two-stream Attention Progressive Semantically-Nested Network	Two nuclear medicine physicians
Yue Y. et al. [32] (2022)	CT, PET	0.72 ± 0.02/ 11.87 ± 4.20	Esophageal cancer (166)	Two-Stream 3D PSNN	3D Progressive Semantically Nested Network	Two experienced nuclear medicine physicians
Lou X. et al. [33] (2024)	CT	0.72 ± 19.18/ 3.98 ± 3.01	Esophageal Cancer (124)	Modified U-Net architecture	Enhanced attention and frequency-aware U-Net variant optimized for advanced feature extraction and fusion	Three radiation oncologists

Abbreviations: PSNN: Progressive semantically nested network, Global and Local Attention U-Net (GloD-LoATU-Net), Not Available (N/A).

### 3.3. Abdomen/Pelvis

An overview of the studies addressing GTV delineation within the abdominal and pelvic region included in this review (n = 17) is provided in Table 3 [34,35,36,37,38,39,40,41,42,43,44,45,46,47,48,49,50]. One publication focused on pancreatic cancer [45], one on hepatocellular carcinoma [36], four on colon carcinoma limited to the rectal/anal region [34,35,38,43], five focused on prostate cancer [39,40,41,42,47], and six on cervical cancer [37,44,46,49,50]. There were no recent publications focusing on GTV delineation in tumors of the upper gastrointestinal tract, ovaries, or the urinary tract and bladder.

The mean sample size across the abdomen/pelvis subset was N = 97 (range: 15–209). The primary imaging modality in most publications was MRI alone (53%) [34,35,41,43,44,45,46,49,50], followed by PET-CT (30%) [38,39,40,42,47]. Three papers used CT alone for GTV delineation (17%) [36,37,48].

Geng J. et al. published two top-performing papers, both achieving high DSC, using a DpnU-Net-based deep learning framework for GTV segmentation, with DSC = 0.87 ± 0.07 in both studies [34,35]. The HD values were 4.07 ± 1.67 (August) and 5.79 ± 3.00 (September). In the August study (141 patients), two separate DpnU-Net models were trained using MRI for GTV segmentation and CT for clinical target volume (CTV) segmentation, respectively. The September study (88 patients) expanded this by using both MRI and CT in a three-stage process. Stage 1 predicted GTV-MRI from MRI, Stage 2 generated intermediate GTV-CT from CT, and Stage 3 refined the contours by registering the MRI-derived GTV with CT, highlighting the framework’s ability to handle multimodal segmentation tasks effectively.

All the publications disclosed their data sources, demonstrating transparency in data acquisition. Apart from Ghezzo et al. [47], all papers reported on their data split, highlighting a commitment to methodological transparency. Seven of the publications reported using an external dataset. It is noteworthy that only one of these papers was prior to 2023 (Kostyszyn) [37,39,40,41,42,46,47]. More than two-thirds of the publications (88%) reported details about the delineation staff, including the number of staff members and their educational qualifications [34,35,36,37,38,40,41,42,44,45,46,47,48,49,50].

The abdomen subset demonstrated the highest overall adherence, with an average of 81.7% for CLAIM and 86.5% for TRIPOD, resulting in an overall adherence of 84.1% (Figure 2). This highlights consistent reporting practices in studies focused on the abdominal region. The mean DSC outcomes of the examined publications was 0.77 with an IQR of 0.11 (range: 0.62–0.87) and the HD measurements derived had a mean of 7.53 mm, and an IQR of 3.47 mm (range: 2.77–20.44 mm). Figure 3 and Figure 4 summarize the DSC and HD outcomes. 

**Table 3:** Abdomen/pelvis publications [43].

Author (Year)	Modality	DSC/HD	Cancer Type (N)	Model	Architecture	Delineation Staff
Geng J et al. [34] (2023) (August)	MRI	0.87 ± 0.07/ 4.07 ± 1.67	Rectal Cancer (141)	DpuU-Net	U-Net with dual-path-network modules (DPN92).	Eight radiation oncologists
Geng J. et al. [35] (2023) (September)	MRI	0.87 ± 0.07/ 5.79 ± 3.00	Rectal Cancer (88)	DpuU-Net	U-Net with dual-path-network modules (DPN92).	Two oncologists
Yang Z. et al. [36] (2023)	(4D)-CT	0.86 ± 0.08/ 5.14 ± 3.34	Hepatocellular carcinoma (26)	Spatial-temporal dual path U-Net	Dual-path network with spatial-temporal features, and a feature fusion module	Radiation oncologist
Peng H. et al. [37] (2024)	CT	0.84 ± 0.07/ 6.58 ± 5.97	Cervical Cancer (71)	MDSSL 3D-U-Net	Multi-decoder and semi-supervised learning (MDSSL)	Radiation oncologists
Groendahl A. et al. [38] (2022)	CT, PET	0.83 ± 0.08/ 7.07 ± 4.43	Anal squamous cell carcinoma (36)	2D U-NET	U-NET	One oncologist, one radiologist.
Kostyszyn D. et al. [39] (2020)	CT, PET	0.83/ 4.12	Prostate cancer (209)	3D U-NET	U-NET	N/A
Holzschuh J.C. et al. [40] (2023)	CT, PET	0.82 ± 0.07/ 3.30 ± 1.96	Prostate Cancer (52)	3D-U-Net	3D-U-Net with decoder and encoder consisting of 3 layers	Two readers (radiation oncology, radiology or nuclear medicine)
Rajendrang P. et al. [41] (2024)	MRI	0.81 ± 0.10/ 9.86 ± 9.77	Prostate Cancer (133)	Medformer (w. LAVE)	Dual-channel 3D Swin Transformer backbone with visual-language attention and a CNN-based decoder	Radiation oncologist and professional trainee
Holzschuh J.C. et al. [42] (2024)	CT, PET	0.76/ 1.73	Prostate Cancer (161)	nn-U-Net	Dynamically configuration based on input, without fixed backbone.	Two radiation oncologists
Wang J. et al. [43] (2018)	MRI	0.74 ± 0.14/ 20.44 ± 13.35	Rectal cancer (93)	2D U-NET	U-NET	N/A
Outeiral R. et al. [44] (2023)	MRI	0.73/ 6.80	Cervical cancer (195)	3D nn-U-NET	nn-U-NET	One radiation oncologist
Liang Y. et al. [45] (2020)	MRI	0.73 ± 0.09/ 8.11 ± 4.09	Pancreas cancer (56)	Square-window based convolutional neural network	Custom CNN	One oncologist, one radiologist.
Rouhi R. et al. [46] (2024)	MRI	0.72 ± 0.16/ 14.6 ± 9.0	Cervical Cancer (166)	SegResNet	Asymmetrically larger encoder using ResNet blocks, strided convolutions, and a decoder with skip connections	Two radiation oncologists
Ghezzo S. et al. [47] (2023)	CT, PET	0.71 ± 0.19/ N/A	Prostate cancer (85)	3D U-NET (Kostyszyn D. et al. [39])	U-NET	Two nuclear medicine physicians
Chang, JH. et al. [48] (2021)	CT	0.71/ N/A	Cervical cancer (51)	3D U-NET + Long Short-Term Memory	3D U-NET + Long Short-Term Memory	One radiation oncologist
Breto A. et al. [49] (2022)	MRI	0.67/ 2.77 ± 1.73	Cervical cancer (15)	Mask R-CNN	Faster R-CNN (ImageNet) + segmentation	One radiation oncologist
Yoganathan S. et al. [50] (2022)	MRI	0.62 ± 0.14/ 6.83 ± 2.89	Cervical cancer (71)	2.5D DeepLabv3+	ResNet50, InceptionResNetv2	One radiation oncologist

Abbreviations: Not Available (N/A), Neural Network U-Net (nn-U-NET), Convolutional Neural Network (CNN), Region-Based Convolutional Neural Network (R-CNN), Residual Networks 50 (ResNet50), Inception-Residual Network v2 (InceptionResNetV2).

### 3.4. Soft TISSUE and Bone

Four papers aiming to delineate the GTV in soft tissue or bone were included, as listed in Table 4 [51,52,53,54]. The DSC and HD overview is reported in Figure 3 and Figure 4. These publications focus, respectively, on sarcomas (including soft tissue sarcomas, bone sarcomas, and chondromas) and oligo-metastases from primary NSCLC. Two papers proposed solely using CT as the preferred imaging modality for soft tissue and bone sarcoma, and sacral chondroma [52,53]. The remaining two papers using CT in combination with PET for NSCLC bone metastasis [54] or solely relied on MRI for soft tissue sarcoma [51].

Peeken J. C. et al. achieved the best segmentation performance for both DSC and HD using an MRI-based model (DSC = 0.88 ± 0.04, HD = 12.0 ± 4.3), with the largest cohort (244 patients; 157 training and 87 independent external test) [51]. The authors provided detailed cohort statistics, including TNM classification, grading, and AJCC staging. They proposed a modified 3D U-Net featuring an encoder–decoder structure with multi-head self-attention at the bottleneck to improve spatial awareness beyond convolutional limitations. The final architecture was validated on an independent external dataset (n = 87).

All papers disclosed their data sources and delineation references and had a mean sample size of 71 (range: 15–209). The mean DSC outcome of the examined publications in the soft tissue and bone subset was 0.80 with an IQR of 0.07 (range 0.62–0.88), and the HD measurements derived had a mean of 14.20 mm, and an IQR of 2.21 (range: 12.0–16.43). The subset exhibited an adherence to CLAIM with 77.25% and 86.25% for TRIPOD, yielding an overall adherence of 81.75% (Figure 2). This disparity suggests variability in how well studies related to soft tissue or bone align with these reporting guidelines, with stronger adherence to TRIPOD.

**Table 4:** Soft tissue and bone publications.

Author (Year)	Modality	DSC/HD	Cancer Type (N)	Model	Architecture	Delineation Staff
Peeken JC. et al. [51] (2024)	MRI	0.88 ± 0.04/ 12.0 ± 4.3	Soft tissue sarcoma (244)	DLBAS 3D-U-Net	3D U-Net with squeeze and excitation blocks, residual blocks, and multi-head self-attention	Two radiation oncologists
Marin T. et al. [52] (2021)	CT	0.86 ± 0.05/ 16.43 ± 13.26	Soft tissue and bone sarcoma (68)	2.5D U-NET	U-NET	Four radiation oncologists or radiologists
Boussioux L. et al. [53] (2024)	CT	0.85 ± 6.4/ NA	Sacral chordoma (48)	Residual 3D U-Net	Optimal ensemble of residual 3D U-Net	One radiologist
Nigam R. et al. [54] (2023)	CT, PET	0.63 ± 0.12/ NA	NSCLC Bone metastasis (9)	Auto segmentation on SUV thresholding	Custom PET/CT segmentation pipeline	One radiation oncologist

Abbreviations: Not Available (N/A).

## 4. Discussion

This systematic review aimed to outline and critically appraise the recent literature on AI-based automatic delineation of gross tumor volume in radiological images of tumors of the thoracic, abdominal, and pelvic cavity as well as soft tissue and bone. Four main findings were highlighted. Firstly, the diagnostic performance from the use of artificial intelligence ranged 0.62–0.92 in dice similarity coefficient (DSC) and 1.33–47.10 mm in Hausdorff distance (HD). Secondly, for all four topological areas of interest, we observed the highest DSC ranging between 0.86 and 0.92 [7,23,34,51]. Thirdly, the most used architecture for all publications was the U-NET (56.25%), which was also used for three of the top performing algorithms [23,34,51]. Fourthly, the lowest Hausdorff distance and highest dice similarity coefficient observed in three of the subsets (esophagus, abdomen and pelvis, soft tissue, and bone) were also found using a U-NET [23,34,51].

For three of the top-performing proposed methods in the thorax, esophagus, and abdomen subset, time measurements were provided [7,23,34]. Kunkyab et al. and Geng et al. both obtained their automated GTV delineation after 12 s in comparison to the reported manual annotation time of 10–15 min [7,34]. Zhang S. et al. showcased significant improvement in delineation for 6/12 radiologists, as well as reducing the inter- and intra-reader variability by 37.4% and 55.2%, respectively. In addition, the author also reported being able to reduce the annotation time by 77.6% (9.73 to 2.18 min) [23].

The best performance with regards to DSC and HD was obtained by Kunkyab T. et al. (DSC = 0.92, HD = 1.33), utilizing CNN with multi-resolution input and a transformers module [7]. This method shares some similarities with a U-NET in the sense of having an encoder–decoder architecture, both skipping connections to preserve spatial information, and both aiming to conduct multi-scale feature extraction. Kunkyab T. et al.’s proposed method, however, differs in using a parallel dual-branch (deep and shallow) to process different image resolutions. They proceed to integrate a deformable self-attention transformer to enhance region specific focus, deploy a multi-scale feature pyramid network to leverage hierarchical features, and integrate residual blocks in the decoder to refine the segmentation details.

### 4.1. CLAIM and TRIPOD Assessment

Adherence to the CLAIM and TRIPOD guidelines varied across topological areas. These differences might partly reflect the number of studies in each category. The average adherence for TRIPOD was observed to be higher (83.83%) in comparison to CLAIM (79.35). The difference for the adherence between the two quality assessments was, however, not statistically significantly different (normal distribution, *t*-test, *p*-value = 0.008). For esophagus, abdomen, and soft tissue and bone, the overall average adherence to both assessments was above 80% (83.2%, 84.1%, 81.8%). The subset for thorax publications differed with an average adherence of 77.3%, however, not significantly (Kruskal–Wallis chi-squared = 3, degrees of freedom = 3, *p*-value = 0.39).

### 4.2. Clinical Relevance

The diverse array of tumor delineation methodologies across abdominal and thoracic studies prompts a crucial exploration of their clinical relevance. The variability in imaging modalities and algorithmic strategies signifies the adaptability of these methodologies to the heterogeneous clinical landscape.

There are sparse to no publications regarding GTV segmentation of tumors of the mediastinal cavity, upper GI tract, and abdominal cavity (e.g., gastric or ovarian tumors), which we interpreted in light of the segmentation difficulties that these tumors pose given their irregular and infiltrative nature as well as their association with vital cardiac and vascular structures. This, coupled with the variability of manual GTV segmentations in such anatomical districts, should prompt a discussion for clearer and more standardized guidelines for GTV delineation. To this extent, it might be speculated that the lack of clear clinical standards makes the development of reliable algorithms in this space hard if not impossible.

The flexibility of GTV delineation instructions is also reflected in the imaging modalities used for algorithmic segmentation (CT, MRI, PET). Although it must be appreciated that no one-size-fits-all solution is possible due to the diverse biological nature and hence the radiological appearance of such tumors, which also may differ depending on the imaging modality, as well as logistical factors due to different imaging availabilities of different centers, more research on the best imaging modality or modalities for automatic GTV delineation for each tumor type is required before translating this technology to real-life clinical scenarios.

### 4.3. Methodical Considerations

This systematic review boasts several strengths contributing to its robustness. All selected papers leveraged data from diverse imaging modalities such as CT, PET, and MRI, ensuring a comprehensive exploration of tumor delineation methods. Notably, there were no restrictions on patient numbers or algorithmic architectures, fostering inclusivity and enhancing the generalizability of findings. Assessment for bias and reporting adherence using the TRIPOD and CLAIM checklist was conducted in every paper, ensuring transparency and reliability. The review’s criterion, encompassing all cancer types treatable with radiotherapy and requiring GTV delineation, guarantees relevance across varied clinical scenarios.

Although the exclusion of non-peer-reviewed papers may narrow the results reported in this review, it guarantees a rigorous revision of the methods and ethical standards of the reported results. Additionally, our evaluation focused mainly on DSC and HD, which are arguably the most used metrics in the field, making studies comparable. Nevertheless, it is possible that other interesting performance metrics were overlooked. Variability in study design introduces heterogeneity, complicating direct comparisons. Diverse algorithmic parameters and the inclusion of different cancer types and imaging modalities may challenge the identification of optimal approaches. The absence of standardization across studies poses challenges in synthesizing cohesive conclusions. Incomplete reporting in some studies adds complexity, potentially influencing the review’s overall robustness.

### 4.4. Future Directions for Research

Furthermore, an in-depth exploration of the interpretability of algorithms used in tumor delineation is crucial for gaining clinician trust and facilitating clinical adoption. Future research should further address the interpretability of algorithmic outputs, which is seldom assessed in validation studies [55]. Additionally, a more extensive exploration of algorithm robustness in the face of diverse patient populations, including variations in age, sex, comorbidities and scanning protocols as well as other possible sources of bias is essential for ensuring equitable and effective clinical applications. Exploring the integration of multimodal imaging data, combining information from CT, MRI, and PET scans, could be a promising avenue. Investigating how algorithms can effectively leverage complementary data from various modalities may enhance the accuracy and robustness of tumor delineation, particularly in complex cases involving multiple anatomical structures.

Future research could focus on the development and validation of standardized protocols for tumor delineation across different anatomical regions and possibly different tumor types. Such consensus guidelines for standardized imaging acquisition, processing, and algorithmic implementation could enhance comparability and reproducibility across studies and ensure consistent and reliable performance across diverse clinical scenarios. Furthermore, investigating the feasibility and accuracy of real-time tumor delineation during imaging procedures could have significant implications for adaptive radiotherapy and interventions, where timely and precise delineation is critical.

## Figures and Tables

**Table 1:** Thorax Publications.

Author (Year)	Modality	DSC/HD	Cancer Type (N Patients)	Model	Backbone	Delineation STAFF
Kunkyab T. et al. [7] (2024)	CT	0.92 1.33	Lung Cancer (676)	Co-ReTr	CNN with multi resolution input, and Transformers module	Radiation oncologist
Wang S. et al. [8] (2022)	CT, PET	0.85 ± 0.05/ 8.53 ± 3.79	NSCLC (280)	3D CNN Dual-Modality Network	Independent convolution for PET/CT and encoder-decoder architecture	Four radiation oncologists
Xie H. et al. [9] (2022)	CT	0.84 ± 0.0/ 8.11 ±3.43	Lung cancer (127)	TransResSEU-NET 2.5D	3D U-NET with 2D and 3D Res-SE Modules	One radiation oncologist, two radiotherapists
Cui Y. et al. [10] (2021)	CT	0.83 ± 0.07/ 4.57 ± 2.44	NSCLC (192)	Dense V-Networks	Combination of DenseNet and V-Network Structures	Two radiation oncologists
Zhang G. et al. [11] (2022)	CT	0.83 ± 0.10/ 4.02 ± 0.15	Lung Cancer (871)	I-3D DenseU-NET	Nested Dense Skip Connection between Encoder and Decoder Blocks	One radiation oncologist
Talebi P. et al. [12] (2022)	(4D-) CT	0.83 ± 0.13 3.73 ± 0.99	NSCLC (20)	3D-U-Net w. attention module	3D-U-Net with an added attention module	One radiation oncologist
Skett S. et al. [13] (2024)	CT	0.80 ± 0.10/ 10.5 ± 7.3	Lung Cancer (379)	nnU-Net	Anchor-point-based post- processing	Two oncologists
Kawata Y. et al. [14] (2017)	CT, PET	0.79 ± 0.06/ N/A	NSCLC (16)	Automated ML Framework for GTV Segmentation	Pixel-based ML Techniques: FCM, ANN, SVM	Two radiation oncologists
Ikushima K. et al. [15] (2017)	CT, PET	0.77/ N/A	Lung cancer (14)	PET/CT and Diagnostic CT Registration	SVM with Gaussian kernel for classification	Two radiation oncologists
Ma Y. et al. [16] (2022)	CT	0.74 ± 0.15/ 28.23 ± 34.87	Lung cancer (70)	GruU-NET-add	Convolutional GRU-based 3D U-NET	One radiation oncologist
Yu X. et al. [17] (2022)	CT	0.73/ 21.39	Stage III NSCLC (214)	3D ResSE-U-NET	3D U-NET with Residual and SE Blocks	Radiation oncologist
Gan W. et al. [18] (2021)	CT	0.72 ± 0.10/ 21.73 ± 13.30	Lung cancer (260)	Hybrid 2D + 3D CNN	V-Net for 3D CNN; Dense Blocks for 2D CNN	Two radiation oncologists
Wong J. et al. [19] (2021)	CT	0.71 ± 0.19/ 5.23	Lung cancer (96)	Limbus Contour v1.0.22	U-NET	One radiation oncologist
Thomas T. et al. [20] (2017)	CT, PET	0.71/ 8.10	NSCLC (9)	Improved GrowCut	GrowCut Algorithm	N/A
Cheng D. et al. [21] (2020)	CT	0.66/ N/A	NSCLC (25)	Random Walks Algorithm	Graph-based algorithm	One clinical oncologist

Abbreviations: Artificial Neural Network (ANN), Convolutional Neural Network (CNN), Fuzzy C-Means (FCM), Gross Tumor Volume (GTV), Gated Recurrent Unit-based U-NET (Gru-based U-NET), Machine Learning (ML), Not Available (N/A), Residual Squeeze-and-Excitation (Res-SE), Squeeze-and-Excitation Blocks (SE-Blocks), Support Vector Machine (SVM), Transfer Learning with Residual Networks (TransRes).

## Data Availability

Not applicable.
